# (*E*)-2-({2-[(*E*)-(Hy­droxy­imino)­meth­yl]phen­oxy}meth­yl)-3-*o*-tolyl­acrylonitrile

**DOI:** 10.1107/S1600536812001481

**Published:** 2012-01-21

**Authors:** E. Govindan, J. Srinivasan, M. Bakthadoss, A. SubbiahPandi

**Affiliations:** a Department of Physics, Presidency College (Autonomous), Chennai 600 005, India; b Department of Organic Chemistry, University of Madras, Guindy Campus, Chennai 600 025, India

## Abstract

In the title compound, C_18_H_16_N_2_O_2_, the dihedral angle between the mean planes through the two benzene rings is 56.8 (6)°. The enoate group assumes an extended conformation. The hy­droxy­ethanimine group is essentially coplanar with the benzene ring, the largest deviation from the mean plane being 0.047 (1) Å for the hy­droxy­imino O atom. In the crystal, the mol­ecules are linked into cyclic centrosymmetric dimers with *R*_2_^2^(6) motifs *via* O—H⋯N hydrogen bonds.

## Related literature

For the use of 2-cyano­acrylates and oximes as agrochemicals, see: Zhang *et al.* (2009 ▶). For the use of oximes as chelating ligands in coordination and analytical chemistry, see: Chaudhuri *et al.* (2003 ▶). For a related structure, see: Govindan *et al.* (2011 ▶).
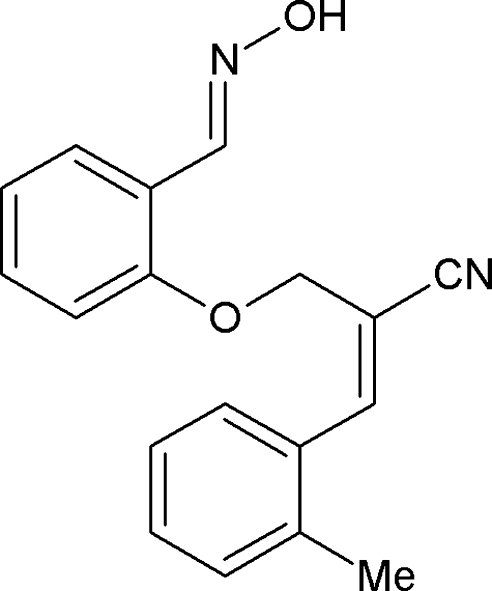


## Experimental

### 

#### Crystal data

C_18_H_16_N_2_O_2_
*M* *_r_* = 292.33
Triclinic, 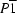

*a* = 7.0214 (2) Å
*b* = 10.5094 (3) Å
*c* = 10.8854 (3) Å
α = 97.636 (1)°
β = 95.953 (1)°
γ = 99.642 (1)°
*V* = 778.32 (4) Å^3^
*Z* = 2
Mo *K*α radiation
μ = 0.08 mm^−1^
*T* = 293 K
0.25 × 0.22 × 0.19 mm

#### Data collection

Bruker APEXII CCD area-detector diffractometer
Absorption correction: multi-scan (*SADABS*; Sheldrick, 1996 ▶) *T*_min_ = 0.978, *T*_max_ = 0.983
21189 measured reflections
5557 independent reflections
3825 reflections with *I* > 2σ(*I*)
*R*_int_ = 0.029

#### Refinement

*R*[*F* ^2^ > 2σ(*F* ^2^)] = 0.050
*wR*(*F* ^2^) = 0.163
*S* = 1.06
5557 reflections
201 parameters
H-atom parameters constrained
Δρ_max_ = 0.26 e Å^−3^
Δρ_min_ = −0.19 e Å^−3^

### 

Data collection: *APEX2* (Bruker, 2004 ▶); cell refinement: *SAINT* (Bruker, 2004 ▶); data reduction: *SAINT*; program(s) used to solve structure: *SHELXS97* (Sheldrick, 2008 ▶); program(s) used to refine structure: *SHELXL97* (Sheldrick, 2008 ▶); molecular graphics: *ORTEP-3* (Farrugia, 1997 ▶); software used to prepare material for publication: *SHELXL97* and *PLATON* (Spek, 2009 ▶).

## Supplementary Material

Crystal structure: contains datablock(s) global, I. DOI: 10.1107/S1600536812001481/qm2049sup1.cif

Structure factors: contains datablock(s) I. DOI: 10.1107/S1600536812001481/qm2049Isup2.hkl

Supplementary material file. DOI: 10.1107/S1600536812001481/qm2049Isup3.cml

Additional supplementary materials:  crystallographic information; 3D view; checkCIF report

## Figures and Tables

**Table 1:** Hydrogen-bond geometry (Å, °)

*D*—H⋯*A*	*D*—H	H⋯*A*	*D*⋯*A*	*D*—H⋯*A*
O1—H1*A*⋯N1^i^	0.82	2.07	2.7962 (13)	147

Symmetry code: (i) 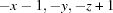.

**Fig. 1.**
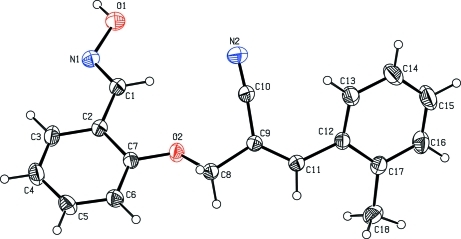
View of the title molecule with the atom labelling scheme. The displacement ellipsoids are drawn at the 30% probability level while the H atoms are shown as small spheres of arbitrary radii.

**Fig. 2.**
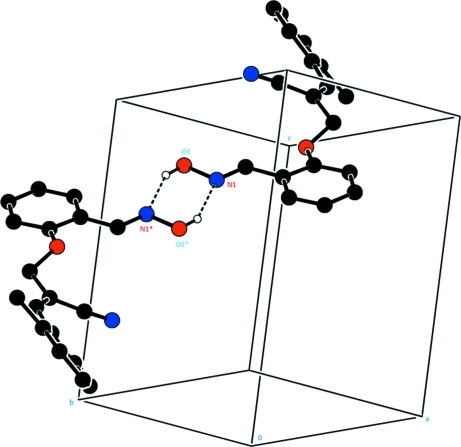
The crystal structure showing the centrosymmetric hydrogen bond motif R22(6). For the sake of clarity, the H atoms not involved in the motif have been omitted. The atoms marked with an asterisk (*) are at the symmetry position (-1 - x, -y, 1 - z). The dashed lines indicate the hydrogen bonds.

## supplementary crystallographic information

### Comment

Recently, 2-cyanoacrylates have been extensively used as agrochemicals because of their unique mechanism of action and good environmental profiles (Zhang *et al.*, 2009). Oximes are a classical type of chelating ligands which are widely used in coordination and analytical chemistry (Chaudhuri, 2003). Against this background, and in order to obtain detailed information on molecular conformations in the solid state, an X-ray study of the title compound was carried out.

X-Ray analysis confirms the molecular structure and atom connectivity as illustrated in Fig. 1. The bond lengths and angles in (Fig. 1) agree with those observed in other tolylacrylonitile derivatives (Govindan *et al.*, 2011). The whole molecule is not planar as the dihedral angle between the two phenyl rings is 56.8 (6)°, The oxime group having the C=N forming an E configuration. The hydroxyethanimine group is essentially coplanar with the benzene ring, the largest deviation from the mean plane of the hydroxyethanimine [C=N—OH] group is 0.047 (1) Å. for the O1 atom.

The enoate group assumes an extended conformation as can be seen from torsion angles C2—C1—N1—O1 [177.9 (2)°] and C1—C2—C3—C4 [-177.1 (2) °]. The atom C15 in the molecule (*x*,*y*,*z*) donate one proton to atom O1 of the molecule at (-1 - *x*,-*y*,1 - *z*) forming a C(6) chain along *b* axis. The hydroxyethanimine group in the molecules are linked into cyclic centrosymmetric dimers *via* O—H···N hydrogen bonds with the motif *R*^2^_2_(6) (Fig. 2). In addition to van der Waals interaction, the crystal packing is stabilized by C—H···O interactions.

### Experimental

To a stirred solution of (*E*)-2-((2-formylphenoxy)methyl)-3-*o*- tolylacrylonitrile (4 mmol) in 10 ml of EtOH/H_2_O mixture (1:1) was added NH_2_OH.HCl (6 mmol) in the presence of 50% NaOH at room temperature. Then the reaction mixture was allowed to stir at room temperature for 1.5 h. After completion of the reaction, solvent was removed and the crude mass was diluted with water (15 ml) and extracted with ethyl acetate (3 *x* 15 ml). The combined organic layer was washed with brine (2 *x* 10 ml) and dried over anhydrous Na_2_SO_4_ and then evaporated under reduced pressure to obtain (2E)-2-((2-((Hydroxyimino)methyl) phenoxy)methyl)-3-*o*-tolylacrylonitrile as a colourless solid.

### Refinement

All H atoms were fixed geometrically and allowed to ride on their parent C atoms, with C—H distances fixed in the range 0.93–0.97 Å with *U*_iso_(H) = 1.5*U*_eq_(C) for methyl H 1.2*U*_eq_(C) for other H atoms.

### Crystal data

**Table:**

C_18_H_16_N_2_O_2_	*Z* = 2
*M**_r_* = 292.33	*F*(000) = 308
Triclinic, *P*1	*D*_x_ = 1.247 Mg m^−^^3^
Hall symbol: -P 1	Mo *K*α radiation, λ = 0.71073 Å
*a* = 7.0214 (2) Å	Cell parameters from 5557 reflections
*b* = 10.5094 (3) Å	θ = 1.9°
*c* = 10.8854 (3) Å	µ = 0.08 mm^−^^1^
α = 97.636 (1)°	*T* = 293 K
β = 95.953 (1)°	Block, white crystalline
γ = 99.642 (1)°	0.25 × 0.22 × 0.19 mm
*V* = 778.32 (4) Å^3^	

### Data collection

**Table:**

Bruker APEXII CCD area-detector diffractometer	5557 independent reflections
Radiation source: fine-focus sealed tube	3825 reflections with *I* > 2σ(*I*)
graphite	*R*_int_ = 0.029
ω and φ scans	θ_max_ = 34.6°, θ_min_ = 1.9°
Absorption correction: multi-scan (*SADABS*; Sheldrick, 1996)	*h* = −10→11
*T*_min_ = 0.978, *T*_max_ = 0.983	*k* = −16→15
21189 measured reflections	*l* = −16→16

### Refinement

**Table:**

Refinement on *F*^2^	Primary atom site location: structure-invariant direct methods
Least-squares matrix: full	Secondary atom site location: difference Fourier map
*R*[*F*^2^ > 2σ(*F*^2^)] = 0.050	Hydrogen site location: inferred from neighbouring sites
*wR*(*F*^2^) = 0.163	H-atom parameters constrained
*S* = 1.06	*w* = 1/[σ^2^(*F*_o_^2^) + (0.0845*P*)^2^ + 0.0621*P*] where *P* = (*F*_o_^2^ + 2*F*_c_^2^)/3
5557 reflections	(Δ/σ)_max_ < 0.001
201 parameters	Δρ_max_ = 0.26 e Å^−^^3^
0 restraints	Δρ_min_ = −0.18 e Å^−^^3^

### Special details

**Table:**

Geometry. All e.s.d.'s (except the e.s.d. in the dihedral angle between two l.s. planes) are estimated using the full covariance matrix. The cell e.s.d.'s are taken into account individually in the estimation of e.s.d.'s in distances, angles and torsion angles; correlations between e.s.d.'s in cell parameters are only used when they are defined by crystal symmetry. An approximate (isotropic) treatment of cell e.s.d.'s is used for estimating e.s.d.'s involving l.s. planes.
Refinement. Refinement of *F*^2^ against ALL reflections. The weighted *R*-factor *wR* and goodness of fit *S* are based on *F*^2^, conventional *R*-factors *R* are based on *F*, with *F* set to zero for negative *F*^2^. The threshold expression of *F*^2^ > σ(*F*^2^) is used only for calculating *R*-factors(gt) *etc*. and is not relevant to the choice of reflections for refinement. *R*-factors based on *F*^2^ are statistically about twice as large as those based on *F*, and *R*- factors based on ALL data will be even larger.

### Fractional atomic coordinates and isotropic or equivalent isotropic displacement parameters (Å^2^)

**Table:**

	*x*	*y*	*z*	*U*_iso_*/*U*_eq_	
C1	−0.15593 (16)	0.00826 (11)	0.65422 (11)	0.0458 (3)	
H1	−0.1182	0.0760	0.7210	0.055*	
C2	−0.02936 (15)	−0.08709 (10)	0.63051 (10)	0.0403 (2)	
C3	−0.08597 (19)	−0.20010 (12)	0.54286 (12)	0.0537 (3)	
H3	−0.2068	−0.2148	0.4943	0.064*	
C4	0.0343 (2)	−0.29046 (12)	0.52696 (13)	0.0590 (3)	
H4	−0.0051	−0.3649	0.4674	0.071*	
C5	0.21237 (19)	−0.27051 (12)	0.59921 (12)	0.0536 (3)	
H5	0.2919	−0.3327	0.5897	0.064*	
C6	0.27362 (17)	−0.15902 (11)	0.68554 (11)	0.0465 (3)	
H6	0.3947	−0.1455	0.7337	0.056*	
C7	0.15422 (14)	−0.06683 (10)	0.70047 (9)	0.0381 (2)	
C8	0.34958 (15)	0.05707 (11)	0.88418 (10)	0.0430 (2)	
H8A	0.4795	0.0729	0.8598	0.052*	
H8B	0.3317	−0.0240	0.9187	0.052*	
C9	0.32336 (14)	0.16826 (10)	0.97920 (10)	0.0387 (2)	
C10	0.12321 (15)	0.17173 (11)	0.99392 (11)	0.0467 (3)	
C11	0.47398 (14)	0.25582 (10)	1.04165 (10)	0.0395 (2)	
H11	0.5958	0.2450	1.0203	0.047*	
C12	0.47398 (15)	0.36647 (10)	1.13905 (10)	0.0399 (2)	
C13	0.3269 (2)	0.36659 (13)	1.21596 (12)	0.0552 (3)	
H13	0.2299	0.2930	1.2087	0.066*	
C14	0.3234 (2)	0.47427 (15)	1.30267 (15)	0.0735 (4)	
H14	0.2238	0.4735	1.3527	0.088*	
C15	0.4678 (3)	0.58260 (15)	1.31470 (16)	0.0760 (5)	
H15	0.4642	0.6563	1.3714	0.091*	
C16	0.6170 (2)	0.58189 (12)	1.24310 (14)	0.0609 (3)	
H16	0.7149	0.6554	1.2533	0.073*	
C17	0.62672 (16)	0.47510 (11)	1.15593 (11)	0.0441 (2)	
C18	0.79547 (18)	0.47851 (14)	1.08190 (15)	0.0597 (3)	
H18A	0.8811	0.5616	1.1046	0.090*	
H18B	0.7484	0.4657	0.9943	0.090*	
H18C	0.8651	0.4104	1.0993	0.090*	
N1	−0.31578 (13)	0.00044 (10)	0.58572 (9)	0.0461 (2)	
N2	−0.03874 (16)	0.17076 (12)	0.99832 (14)	0.0698 (4)	
O1	−0.41663 (12)	0.09866 (9)	0.62794 (9)	0.0605 (3)	
H1A	−0.5163	0.0935	0.5797	0.091*	
O2	0.20712 (11)	0.04926 (7)	0.77921 (7)	0.0467 (2)	

### Atomic displacement parameters (Å^2^)

**Table:**

	*U*^11^	*U*^22^	*U*^33^	*U*^12^	*U*^13^	*U*^23^
C1	0.0381 (5)	0.0484 (6)	0.0469 (6)	0.0088 (4)	−0.0016 (4)	−0.0025 (5)
C2	0.0366 (5)	0.0415 (5)	0.0403 (5)	0.0061 (4)	0.0010 (4)	0.0014 (4)
C3	0.0508 (6)	0.0495 (6)	0.0520 (7)	0.0048 (5)	−0.0073 (5)	−0.0076 (5)
C4	0.0703 (8)	0.0439 (6)	0.0553 (7)	0.0082 (6)	0.0010 (6)	−0.0108 (5)
C5	0.0648 (7)	0.0449 (6)	0.0527 (7)	0.0205 (5)	0.0097 (6)	−0.0010 (5)
C6	0.0455 (6)	0.0466 (6)	0.0471 (6)	0.0159 (5)	0.0017 (4)	0.0005 (5)
C7	0.0387 (5)	0.0371 (5)	0.0368 (5)	0.0077 (4)	0.0022 (4)	0.0004 (4)
C8	0.0359 (5)	0.0443 (5)	0.0456 (6)	0.0138 (4)	−0.0041 (4)	−0.0056 (4)
C9	0.0350 (4)	0.0378 (5)	0.0422 (5)	0.0098 (4)	0.0021 (4)	−0.0002 (4)
C10	0.0386 (5)	0.0400 (5)	0.0571 (7)	0.0063 (4)	0.0048 (5)	−0.0061 (5)
C11	0.0360 (5)	0.0395 (5)	0.0415 (5)	0.0082 (4)	0.0023 (4)	0.0015 (4)
C12	0.0402 (5)	0.0369 (5)	0.0403 (5)	0.0070 (4)	0.0004 (4)	0.0008 (4)
C13	0.0579 (7)	0.0501 (6)	0.0516 (7)	−0.0010 (5)	0.0152 (5)	−0.0060 (5)
C14	0.0811 (10)	0.0672 (9)	0.0675 (9)	0.0059 (7)	0.0313 (8)	−0.0141 (7)
C15	0.0977 (12)	0.0526 (8)	0.0692 (9)	0.0061 (7)	0.0200 (8)	−0.0191 (7)
C16	0.0689 (8)	0.0408 (6)	0.0638 (8)	−0.0020 (6)	0.0026 (6)	−0.0049 (5)
C17	0.0426 (5)	0.0390 (5)	0.0477 (6)	0.0057 (4)	−0.0020 (4)	0.0043 (4)
C18	0.0435 (6)	0.0541 (7)	0.0775 (9)	−0.0001 (5)	0.0086 (6)	0.0063 (6)
N1	0.0370 (4)	0.0491 (5)	0.0508 (5)	0.0111 (4)	0.0010 (4)	0.0023 (4)
N2	0.0416 (5)	0.0669 (7)	0.0954 (9)	0.0107 (5)	0.0122 (5)	−0.0110 (6)
O1	0.0460 (5)	0.0644 (6)	0.0689 (6)	0.0224 (4)	−0.0026 (4)	−0.0054 (4)
O2	0.0442 (4)	0.0416 (4)	0.0490 (4)	0.0146 (3)	−0.0114 (3)	−0.0076 (3)

### Geometric parameters (Å, °)

**Table:**

C1—N1	1.2655 (14)	C10—N2	1.1415 (15)
C1—C2	1.4621 (14)	C11—C12	1.4658 (13)
C1—H1	0.9300	C11—H11	0.9300
C2—C3	1.3932 (15)	C12—C13	1.3950 (16)
C2—C7	1.3958 (14)	C12—C17	1.4074 (16)
C3—C4	1.3783 (18)	C13—C14	1.3793 (17)
C3—H3	0.9300	C13—H13	0.9300
C4—C5	1.3751 (19)	C14—C15	1.374 (2)
C4—H4	0.9300	C14—H14	0.9300
C5—C6	1.3768 (16)	C15—C16	1.369 (2)
C5—H5	0.9300	C15—H15	0.9300
C6—C7	1.3880 (14)	C16—C17	1.3859 (16)
C6—H6	0.9300	C16—H16	0.9300
C7—O2	1.3658 (12)	C17—C18	1.4987 (17)
C8—O2	1.4225 (12)	C18—H18A	0.9600
C8—C9	1.5032 (13)	C18—H18B	0.9600
C8—H8A	0.9700	C18—H18C	0.9600
C8—H8B	0.9700	N1—O1	1.4016 (12)
C9—C11	1.3364 (14)	O1—H1A	0.8200
C9—C10	1.4367 (14)		
			
N1—C1—C2	121.33 (10)	C9—C11—C12	129.04 (9)
N1—C1—H1	119.3	C9—C11—H11	115.5
C2—C1—H1	119.3	C12—C11—H11	115.5
C3—C2—C7	118.07 (10)	C13—C12—C17	119.11 (10)
C3—C2—C1	122.78 (10)	C13—C12—C11	121.69 (10)
C7—C2—C1	119.13 (9)	C17—C12—C11	119.20 (10)
C4—C3—C2	121.07 (11)	C14—C13—C12	120.96 (12)
C4—C3—H3	119.5	C14—C13—H13	119.5
C2—C3—H3	119.5	C12—C13—H13	119.5
C5—C4—C3	119.99 (11)	C15—C14—C13	119.68 (13)
C5—C4—H4	120.0	C15—C14—H14	120.2
C3—C4—H4	120.0	C13—C14—H14	120.2
C4—C5—C6	120.34 (11)	C16—C15—C14	119.93 (12)
C4—C5—H5	119.8	C16—C15—H15	120.0
C6—C5—H5	119.8	C14—C15—H15	120.0
C5—C6—C7	119.82 (11)	C15—C16—C17	122.09 (12)
C5—C6—H6	120.1	C15—C16—H16	119.0
C7—C6—H6	120.1	C17—C16—H16	119.0
O2—C7—C6	123.73 (9)	C16—C17—C12	118.08 (11)
O2—C7—C2	115.58 (8)	C16—C17—C18	119.87 (11)
C6—C7—C2	120.66 (10)	C12—C17—C18	122.04 (10)
O2—C8—C9	107.03 (8)	C17—C18—H18A	109.5
O2—C8—H8A	110.3	C17—C18—H18B	109.5
C9—C8—H8A	110.3	H18A—C18—H18B	109.5
O2—C8—H8B	110.3	C17—C18—H18C	109.5
C9—C8—H8B	110.3	H18A—C18—H18C	109.5
H8A—C8—H8B	108.6	H18B—C18—H18C	109.5
C11—C9—C10	124.04 (9)	C1—N1—O1	111.76 (9)
C11—C9—C8	122.27 (9)	N1—O1—H1A	109.5
C10—C9—C8	113.68 (9)	C7—O2—C8	118.07 (8)
N2—C10—C9	175.85 (13)		
			
N1—C1—C2—C3	−8.33 (19)	C8—C9—C11—C12	−177.93 (10)
N1—C1—C2—C7	173.47 (11)	C9—C11—C12—C13	26.68 (19)
C7—C2—C3—C4	1.03 (19)	C9—C11—C12—C17	−153.67 (12)
C1—C2—C3—C4	−177.18 (13)	C17—C12—C13—C14	3.8 (2)
C2—C3—C4—C5	0.8 (2)	C11—C12—C13—C14	−176.50 (13)
C3—C4—C5—C6	−1.6 (2)	C12—C13—C14—C15	−0.7 (3)
C4—C5—C6—C7	0.57 (19)	C13—C14—C15—C16	−1.8 (3)
C5—C6—C7—O2	−176.62 (11)	C14—C15—C16—C17	1.1 (3)
C5—C6—C7—C2	1.26 (18)	C15—C16—C17—C12	2.0 (2)
C3—C2—C7—O2	176.01 (10)	C15—C16—C17—C18	−178.74 (15)
C1—C2—C7—O2	−5.71 (15)	C13—C12—C17—C16	−4.40 (18)
C3—C2—C7—C6	−2.03 (17)	C11—C12—C17—C16	175.94 (11)
C1—C2—C7—C6	176.25 (10)	C13—C12—C17—C18	176.37 (12)
O2—C8—C9—C11	−135.77 (11)	C11—C12—C17—C18	−3.29 (17)
O2—C8—C9—C10	43.22 (13)	C2—C1—N1—O1	177.80 (10)
C11—C9—C10—N2	151.8 (19)	C6—C7—O2—C8	−25.64 (15)
C8—C9—C10—N2	−27 (2)	C2—C7—O2—C8	156.39 (10)
C10—C9—C11—C12	3.19 (19)	C9—C8—O2—C7	−157.61 (9)

### Hydrogen-bond geometry (Å, °)

**Table:**

*D*—H···*A*	*D*—H	H···*A*	*D*···*A*	*D*—H···*A*
O1—H1A···N1^i^	0.82	2.07	2.7962 (13)	147

Symmetry codes: (i) −*x*−1, −*y*, −*z*+1.
